# Response to a COVID-19 Outbreak on a University Campus — Indiana, August 2020

**DOI:** 10.15585/mmwr.mm7004a3

**Published:** 2021-01-29

**Authors:** Mark D. Fox, David C. Bailey, Michael D. Seamon, Marie Lynn Miranda

**Affiliations:** ^1^Saint Joseph County Department of Health, South Bend, Indiana; ^2^Indiana University School of Medicine, South Bend; ^3^University of Notre Dame, Indiana.

Institutions of higher education adopted different approaches for the fall semester 2020 in response to the coronavirus disease 2019 (COVID-19) pandemic. Approximately 45% of colleges and universities implemented online instruction, more than one fourth (27%) provided in-person instruction, and 21% used a hybrid model (*1*). Although CDC has published COVID-19 guidance for institutions of higher education (*2*–*4*), little has been published regarding the response to COVID-19 outbreaks on college and university campuses (*5*). In August 2020, an Indiana university with approximately 12,000 students (including 8,000 undergraduate students, 85% of whom lived on campus) implemented various public health measures to reduce transmission of SARS-CoV-2, the virus that causes COVID-19. Despite these measures, the university experienced an outbreak involving 371 cases during the first few weeks of the fall semester. The majority of cases occurred among undergraduate students living off campus, and several large off-campus gatherings were identified as common sources of exposure. Rather than sending students home, the university switched from in-person to online instruction for undergraduate students and instituted a series of campus restrictions for 2 weeks, during which testing, contact tracing, and isolation and quarantine programs were substantially enhanced, along with educational efforts highlighting the need for strict adherence to the mitigation measures. After 2 weeks, the university implemented a phased return to in-person instruction (with 85% of classes offered in person) and resumption of student life activities. This report describes the outbreak and the data-driven, targeted interventions and rapid escalation of testing, tracing, and isolation measures that enabled the medium-sized university to resume in-person instruction and campus activities. These strategies might prove useful to other colleges and universities responding to campus outbreaks.

## Preparations for Fall Semester

In May 2020, a medium-sized Indiana university announced plans to reopen for in-person instruction for the fall semester. In preparation, the university implemented various public health measures, including rearranging physical infrastructure in high-traffic areas, reducing population density in classrooms and common spaces, enhancing cleaning and disinfection protocols, and requiring masks on campus, including outdoors, when physical distancing of 6 feet could not be maintained. Residence halls maintained usual occupancy levels, although students requesting accommodation for medical reasons were offered individual rooms. The university established an on-campus testing site, identified isolation and quarantine space, hired contact tracers, implemented a daily health check platform (a required online assessment of COVID-19 symptoms and exposures), and developed COVID-19–related data systems (*6*).

Classes began on August 10. The university required preentry SARS-CoV-2 reverse transcription–polymerase chain reaction (RT-PCR) testing for all students 7–10 days before their arrival on campus.* Of the 11,836 students tested, 33 (0.28%) received positive test results and were not allowed on campus until they were cleared to discontinue isolation 10 days after symptom onset or test date (*7*).

Despite these measures, the university experienced an outbreak (defined as an excess of cases compared with the baseline dates of August 3–15) soon after the semester started. To describe the campus outbreak and the university’s response to continue the semester in person, university leaders and a local public health official reviewed university data on daily health checks, testing, contact tracing, isolation, and quarantine. Symptom and testing data, which are combined with university administrative data (e.g., faculty, staff member, or student designation; residence hall; class schedules; and seating charts), were analyzed to estimate symptom prevalence among various subgroups to identify emerging transmission patterns and assist in identifying close contacts. This activity was determined to be public health surveillance as defined in 45 CFR 46.102(l).^†^

## Campus Outbreak and Response

**FIGURE 1:**
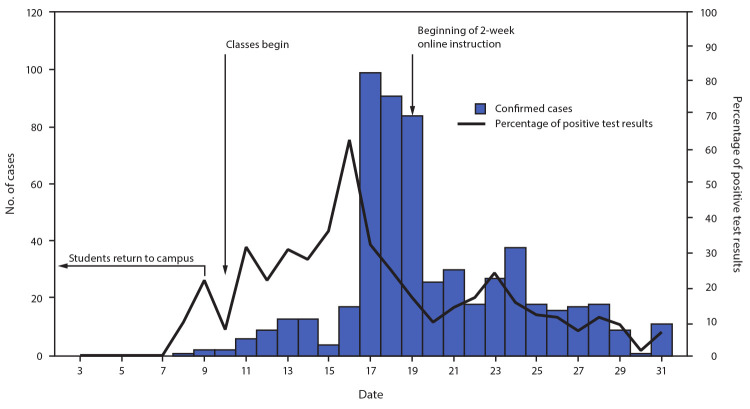
Number of COVID-19 cases confirmed through diagnostic testing,* by test date, and percentage of positive diagnostic test results before and during a COVID-19 outbreak on a university campus — Indiana, August 2020^†^ **Abbreviations:** COVID-19 = coronavirus disease 2019; RT-PCR = reverse transcription–polymerase chain reaction. * Diagnostic tests were ordered for symptomatic persons or close contacts of persons with a confirmed case. A rapid antigen test was performed first, followed by an RT-PCR test if the rapid test was negative. (This figure only includes diagnostic test results and does not include results from screening tests.) ^†^ Student leaders began returning in late July; however, the majority of students returned during August 3–9, after residence halls opened on August 3.

During August 3–15, a total of 56 persons received positive SARS-CoV-2 test results (an average of 4.3 per day, representing 11.7% of all tests performed); 90% of cases were identified through testing of symptomatic persons, with the remainder identified through screening tests of student athletes. During August 16–22, the university experienced an outbreak (Figure 1), with 371 confirmed cases (an average of 26.5 cases per day, representing 15.3% of all tests performed), 355 (96%) of which were in undergraduate students and 13 (3%) in graduate students; 62% of affected undergraduate students lived off campus. One faculty member and two staff members received positive test results. Contact tracing identified several large, off-campus parties where campus masking and physical distancing guidelines were not followed as common sources of exposure for approximately two thirds of cases among undergraduate students.

On August 19, the university implemented a switch to online instruction for all undergraduate classes for a minimum of 2 weeks; graduate and professional classes continued in person. Several temporary campus restrictions were instituted as well, including restricting undergraduate students who lived off campus from the campus (except to access campus health services) and requiring on-campus students to minimize nonessential activities and to remain on campus at all times for at least 2 weeks. Residence halls were restricted to persons who lived or worked in them, student organizations were required to meet remotely, and indoor recreational facilities were temporarily closed. Students were required to eat outside, maintaining 6 feet of distance from others, or in their residence hall rooms, and gatherings were limited to ≤10 persons (both on campus and off campus, although this was difficult to enforce off campus), with mandatory masking and physical distancing. In addition, masks were mandated at all times in all spaces, except in a person’s assigned residence hall room or private office. 

During the 2-week period of online instruction, the university focused on facilitating access to testing; expanding contact tracing, isolation, and quarantine operations; and implementing screening tests for asymptomatic persons, as well as enhancing the data systems to support these measures. Before the outbreak, modifications to the daily health check platform could be made only by the software provider on a set schedule, limiting the ability of the university to respond to changing circumstances. Improvements to this platform facilitated data retrieval, allowing a more detailed view of symptom prevalence and the ability to automate test orders when necessary.

To reduce barriers to testing,^§^ the university increased the test site hours and capacity. Orders for diagnostic testing were automated in response to the presence of primary COVID-19 symptoms (temperature >100.4°F [38°C], new onset of shortness of breath or difficulty breathing, or new loss of sense of taste or smell). Persistent secondary COVID-19 symptoms (minor symptoms, such as headache or rhinorrhea, lasting ≥2 days) or reported close contact with a person with COVID-19 also automatically generated test orders, eliminating the need for clinicians to triage and authorize testing. Rapid antigen tests were used as the front-line diagnostic test because they facilitated rapid isolation and quarantine. Persons with negative antigen test results who were symptomatic or determined to be close contacts received a follow-up RT-PCR test, with results typically available within 36 hours.

The university enhanced contact tracing efforts and redefined workflows to facilitate timely identification and quarantine of close contacts of persons with confirmed COVID-19. During the 2-week outbreak, the contact tracing team expanded from nine full-time staff members to 11 full-time and 13 part-time workers. A new Daily Care and Concern Team was established to ensure that students in isolation and quarantine received meals and other needed resources; this team, consisting of 12 reassigned university staff members and 60 volunteers, also telephoned everyone in isolation and quarantine daily to monitor for worsening symptoms. The university initially reserved 250 beds for isolation and quarantine purposes, increasing to 1,007 beds during the surge of cases, through use of apartments and hotels on or adjacent to campus. During August 16–29, a total of 1,250 students were placed in isolation and quarantine; students with access to adequate facilities (i.e., allowed them to sleep separately from others and had access to a private bathroom) were permitted to isolate or quarantine off campus. In addition to the 371 cases identified during the first week of the outbreak, another 160 were identified during the second week of the outbreak. Slightly more than one half (52%) of the newly positive test results were in persons who were already in quarantine. Among 802 persons in quarantine during this 2-week period, 83 (10.3%) ultimately received a positive SARS-CoV-2 test result. In the week after the return to in-person instruction, an average of four cases per day were identified.

An enhanced communications campaign was created to underscore the importance of adhering to campus public health protocols. The campaign included e-mails from university administrators and campus leaders, video messages, and virtual town hall meetings. The proportion of e-mails sent to the student e-mail distribution list that were viewed (a measure of the reach of these education efforts) was 84.1%.

## Implementation of Screening

**FIGURE 2:**
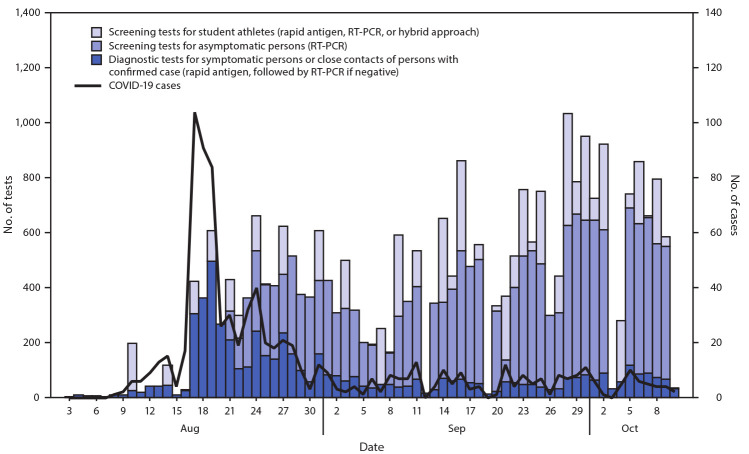
Number of COVID-19 tests performed, by test indication, and number of COVID-19 cases before, during, and after a COVID-19 outbreak on a university campus — Indiana, August–October 2020 **Abbreviations:** COVID-19 = coronavirus disease 2019; RT-PCR = reverse transcription–polymerase chain reaction.

Before the outbreak, testing had been focused on symptomatic persons; routine screening tests were performed for student athletes but had not yet been implemented for the broader university community. After recognition of the outbreak, the university began screening asymptomatic persons with RT-PCR tests on specimens collected by supervised, self-administered nasal swabs. The capacity for screening testing increased throughout the semester (Figure 2). Each round of screening was informed by the previous round and by diagnostic testing trends, using a Bayesian stratified, staggered-entry rotating cohort design (*8*). Persons were grouped into various cohorts (e.g., those who lived in a particular residence hall), and a fraction of each cohort was sampled in each round. Some screening slots were reserved for the evaluation of persons in areas with increased risk for transmission (i.e., potential hotspots). The team responsible for the general campus screening strategy was able to adapt based on disease prevalence in certain groups, such as by college, membership group (club or team), residence hall, or even the floor or wing of a residence hall, to allow oversampling. Diagnostic testing, which was performed for symptomatic persons and for close contacts of persons with SARS-CoV-2 infection, increased from an average of 17.9 tests per day before the outbreak to 208.4 per day during the 2-week outbreak. Likewise, screening increased to 205 tests per day by the end of August.

Based on the decreasing case numbers, increased testing capacity, and enhanced ability to analyze and respond based on data, lower-level undergraduate classes resumed on September 2 (2 weeks after online instruction began), with upper-level undergraduate classes resuming a few days later. Other campus restrictions were gradually relaxed (e.g., coming to or leaving campus and residence hall visitation), and student activities were phased in over the subsequent 7–10 days; however, the requirement for universal masking remains.

During the week ending October 10, 2020, a total of 3,981 tests were performed (521 diagnostic and 3,460 screening tests; overall, 0.9% of test results were positive). The mean 7-day rolling average was five new cases per day, comparable to the overall incidence in the county at the time.

## Discussion

A COVID-19 outbreak on a university campus is a substantial challenge but was managed on a medium-sized campus while students remained in residence (*5*). Analysis of administrative data (e.g., undergraduate versus graduate students and on-campus versus off-campus students or activities) facilitated identification of potential problems, which was critical to designing a specific, tailored response. The stratified rotating cohort approach to screening that was implemented at the university can be used as an alternative to repeated campuswide testing of all students and might be more feasible for resource-constrained institutions. A swift, marked increase in testing, contact tracing, and isolation measures requires a substantial commitment of physical, personnel, and financial resources, which might not be readily available at all colleges and universities of comparable size. In addition, encouraging student adherence to mitigation strategies as a means to eventually continuing the semester in person was critical to the success of these efforts.

The findings in this report are subject to at least two limitations. First, the daily health check relied on self-reported symptoms, and no consequences were associated with failing to complete the health check. This might have led to an underestimate in the number of cases because symptoms might have gone unrecognized or underreported (and thus automated test orders not generated). Conversely, in the absence of widespread screening, any unrecognized cases could have contributed to further spread on campus. Second, although the university provided an on-campus testing site, persons were also able to obtain testing at other community locations, which might have delayed reporting of results or otherwise affected the university’s ability to respond to cases identified among members of the university community, as well as possibly resulting in an underestimate. This underscores the importance of universities working closely with the local health department to facilitate timely reporting of cases and identification of close contacts.

Immediate, aggressive measures to decrease SARS-CoV-2 transmission through enhanced testing, timely contact tracing, provision of adequate isolation and quarantine space, increased screening of asymptomatic persons, and communication promoting adherence to mitigation strategies can help control COVID-19 outbreaks while minimizing disruptions to in-person instruction. This approach is consistent with recommendations for universities with outbreaks to avoid sending students home to avoid spreading infections into local and other communities (*9*).

**Summary**

**What is already known about this topic?**

Although various implementation strategies for SARS-CoV-2 testing on college and university campuses have been described, little has been published regarding successful responses to COVID-19 outbreaks on campus.

**What is added by this report?**

In response to a COVID-19 outbreak on a university campus in August 2020, rapid implementation of multiple measures, including aggressive testing, tracing, and isolation; enhanced data systems; and communication focused on adherence to mitigation strategies, resulted in a rapid decrease in new cases and allowed in-person learning to resume.

**What are the implications for public health practice?**

Enhanced testing, timely contact tracing, provision of adequate isolation and quarantine space, increased screening of asymptomatic persons, and communication promoting adherence to mitigation strategies can help control COVID-19 outbreaks on college and university campuses while minimizing disruptions to in-person instruction.

## Footnotes

* Students received an at-home nasal self-swabbing kit by express delivery, with a return mailer for the testing facility of a national commercial laboratory, where RT-PCR tests were performed, with results transmitted to the student and to University Health Services.

† Protection of Human Subjects, 45 C.F.R. part 46.

§ Persons with COVID-19 symptoms received testing using the Sofia SARS Antigen Fluorescent Immunoassay (Quidel) rapid antigen test. Those with positive test results were isolated. Those with negative tests were also isolated, pending the results of a follow-up RT-PCR test on a nasal swab specimen, performed by a local commercial laboratory primarily using a Roche platform (https://www.labcorp.com/coronavirus-disease-covid-19/providers).
